# Revision total knee arthroplasty with Charcot-Marie-Tooth disease - a case report

**DOI:** 10.1016/j.ijscr.2025.111734

**Published:** 2025-07-24

**Authors:** Jaad Mahlouly, Julien Wegrzyn, Olivier Guyen, Gilles Dietrich, Arnaud Meylan, Alexander Antoniadis

**Affiliations:** a Department of Orthopaedics and Traumatology, Centre Hospitalier Universitaire Vaudois (CHUV), Lausanne, Switzerland

**Keywords:** Charcot-Marie-Tooth, Total knee arthroplasty revision, Multidirectional instability, TKA periprosthetic bone loss, Rotating-hinged TKA, Case report

## Abstract

**Introduction and importance:**

Charcot-Marie-Tooth (CMT) disease is a rare hereditary neuropathy characterized by progressive distal muscle weakness and altered gait biomechanics. Total knee arthroplasty (TKA) in CMT patients can be challenging, as little is known about long-term outcomes. This case highlights the need for pre-operative planning and structured follow-up of patients with CMT who underwent TKA, as loosening might occur at an early stage.

**Case presentation:**

A 72-year-old woman with advanced CMT disease presented with disabling pain and multidirectional instability ten years after a primary TKA. Radiographic evaluation revealed severe aseptic loosening and extensive metaphyseal bone loss. Revision surgery was performed in a one-stage approach using a rotating-hinge TKA with tibial metaphyseal augmentation. The patient experienced excellent recovery and functional improvement. One-stage revision to a rotating-hinge TKA demonstrated a favourable outcome at 3 years.

**Clinical discussion:**

Patients with CMT may present with multiplanar joint instability and abnormal gait, placing increased stress on standard condylar implants and accelerating loosening. In such cases, standard implants may not be appropriate. Early consideration of stemmed or constrained implants may improve stability and durability. Revision surgery may be further complicated by bone loss and instability, warranting the use of rotating-hinged TKA systems. Close postoperative monitoring is critical to detect complications early in this high-risk population.

**Conclusion:**

CMT-related neuromuscular dysfunction may predispose to early TKA failure. This case underlines the importance of preoperative planning and long-term follow-up. When major bone loss and instability are present, revision to a rotating-hinged TKA offers a reliable and durable solution.

## Highlights

• Considering the use of stems during primary surgical planning could potentially improve implant stability and reduce the risk of early failure in this patient population.
• Close follow-up of CMT patients who underwent TKA is crucial in order to detect complications early.
• In the presence of major bone loss and instability, revision to a rotating-hinged TKA offers a valid result.

## 1 Introduction and importance

Charcot-Marie-Tooth (CMT) disease is a hereditary neurodegenerative sensory-motor disorder affecting the peripheral nervous system with a prevalence of 1 in 2500 people [1]. About fifty genes are related to this disease and are responsible for defects in myelination or axonal conduction which leads to a decrease in the speed or intensity of nerve impulse transmission [2].

Patients suffering from CMT gradually develop peripheral muscle weakness. The associated symptoms are typically a combination of tight Achilles tendon, foot-drop, increased foot supination and failure of plantar flexion [3]. In severe forms, CMT might be associated with knee instability characterized by excessive hyperextension and internal rotation during the stance phase of walking [3]. Weakness of ankle plantar flexors might also be present, and a reliable marker of this is the ‘knee bob sign’ where both knees bob up and down when attempting to stand still [4,5].

General treatment modalities include posture stabilization training and properly fitted ankle-foot orthoses to improve walking control [6,7]. Regarding treatment of CMT patients with concomitant advanced knee osteoarthritis, no clear treatment consensus is available. One option is knee arthrodesis as it has been associated with satisfactory outcomes in patients with similar neurological disorders [8]. The alternative might be a total knee arthroplasty (TKA) but to our knowledge, there are no available data in literature that report outcomes of primary or revision TKA in patients suffering from CMT [6,9].

We hereby present a case of a patient with advanced CMT disease who presented with TKA aseptic loosening associated with extensive metaphyseal bone defects 10 years after index surgery. This case has been reported in line with the SCARE 2025 criteria [10].

## 2 Case presentation

**Fig. 1:**
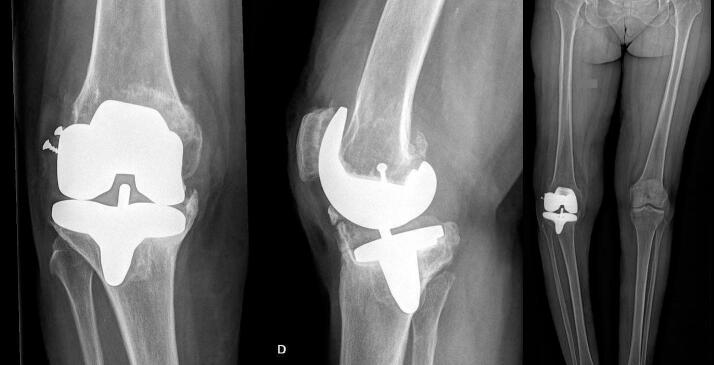
Right knee preoperative (a) anteroposterior; (b) lateral; (c) long leg view radiographs. These are demonstrating massive prosthetic loosening and migration of the components with severe right knee varus (HKA of 162°).

A 72-year-old female patient with advanced CMT disease presenting bilateral distal muscle weakness and requiring the use of bilateral foot-lifting orthosis was referred to our department with a painful and unstable TKA 10 years after primary implantation. During primary surgery, a lateral epicondylar sliding osteotomy was performed for rigid valgus deformity. An unconstrained mobile bearing implant was placed (pfc Sigma RP-F, DePuy Orthopaedics, Warsaw). At that time, the patient had not yet been diagnosed with CMT disease, which may explain the choice of a standard implant. Unfortunately, preoperative and postoperative radiographs having been discarded by the patient, however, the surgeon's postoperative report describes a well-positioned and aligned TKA. The short-term evolution shows functional improvement. However, the patient began experiencing pain 7 years after implantation, which progressively worsened and became disabling by year 10, when she was finally referred to our department. The pain was accompanied by an important coronal and sagittal plane instability. The radiographs showed prosthetic loosening with major bone loss on the femur and tibia. In addition, there was migration of the tibial component and severe varus deformation with a Hip-Knee angle (HKA) of 162° (Fig. 1). The blood work and intra-articular puncture showed no infectious aetiology with no increase in ESR and CRP values (8 mm/h and 1 mg/L respectively).

**Fig. 2:**
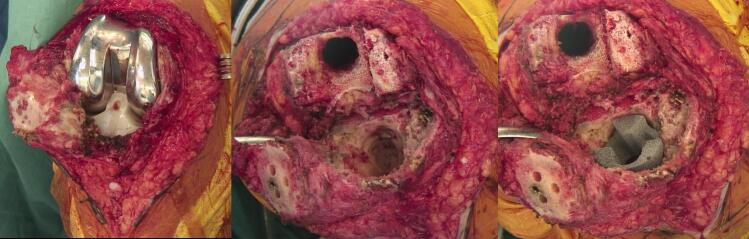
Intraoperative images showing (a) right TKA with metallosis; (b) metaphyseal bone defects after removal of components; (c) augmentation of the proximal tibial metaphysis with tantalum support.

Surgery consisted of a one-stage revision to a rotating-hinge TKA (NexGen RHK, Zimmer, Warsaw). Significant metallosis was present intra-operatively, and careful resection of the periprosthetic fibrosis and total synovectomy was performed (Fig. 2-a). After simple removal of the prosthetic components, metaphyseal bone loss in both the femoral condyles as well as the central and anterior metaphyseal tibial plateau was present. The posterior tibial cortex was still intact. The severity of the bone defects corresponded to a type 2B defect according to the Anderson Orthopaedic Research Institute (AORI) classification.

After debridement of fibrotic tissue, the tibial and distal femoral cuts were refreshed (Fig. 2-b). The control of range of motion, rotation and patellar tracking with trial implants were conclusive. Augmentation of the proximal tibial metaphysis with a tantalum metaphyseal cone (41 × 34 mm) was necessary to compensate extensive bone loss and increase metaphyseal support (Fig. 2-c). Prior to closure, control of stability and mobility was satisfactory with absence of flexion deformity.

**Fig. 3:**
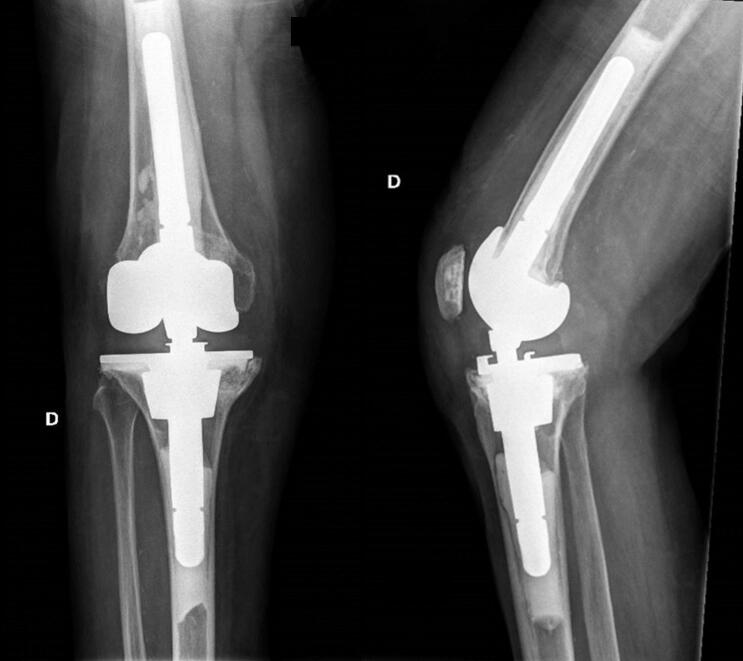
Right knee postoperative (a) anteroposterior; (b) lateral. These are demonstrating a cemented rotating-hinge TKA at 6 weeks post-surgery.

**Fig. 4:**
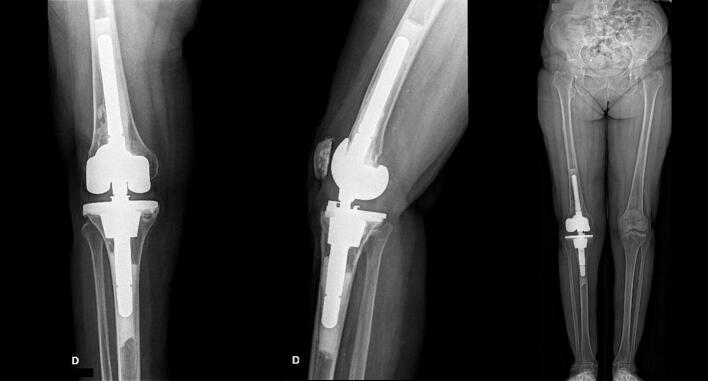
Right knee postoperative (a) anteroposterior; (b) lateral; (c) long leg view radiographs. These are demonstrating a cemented rotating-hinge TKA at 3 years post-surgery with adequate lower right limb alignment (HKA of 180°).

Post-operative radiographic control was adequate (Fig. 3). Post-operative material sonication showed no sign of infection. The patient recovered well and was able to be discharged from the hospital 5 days after surgery. No postoperative complications were reported. At two years post-operatively, the patient showed a favourable evolution with an excellent functional outcome. The radiographic work-up showed no complication at the 3-year follow-up (Fig. 4). Total Knee Society score (KSS) improved significantly from 10/200 to 170/200 after surgery [11] and EQ-5D score improved from 0,52 to 0.75 [12] (Table 1).

**Table 1:** Timeline of clinical events following primary total knee arthroplasty (TKA), including symptom onset, revision procedure, and structured postoperative follow-up. This table illustrates the evolution of the patient's condition and long-term outcome over a 10+ year period.

Time point	Clinical event
Year 0	Primary TKA
Year 7	Onset of pain and progressive instability
Year 10	Presentation with disabling symptoms and revision surgery
6 weeks post-revision	Early post-op follow-up: satisfactory outcome
3 months post-revision	Intermediate follow-up: no complications
6 months post-revision	Functional improvement maintained
1 year post-revision	Stable alignment, excellent KSS and EQ-5D scores
Annual thereafter	Stable clinical and radiographic status maintained

## 3 Clinical discussion and conclusion

To our knowledge, no clear consensus regarding treatment recommendations of patients with CMT suffering from advanced knee osteoarthritis is available [6,9]. As CMT is potentially a progressive condition, aggravation of symptoms such as alteration of gait biomechanics and knee instability [3] might lead to an early loosening. Therefore, it is crucial to follow up these patients closely. To our knowledge this is the first case reporting on revision TKA of a patient suffering from CMT disease.

Considering the altered gait biomechanics of CMT patients, prosthetic components might be under increased strain. During the gait cycle, compensatory mechanisms act in relation to the proximal part of the lower limb to counteract the incorrect supination position of the feet and improve the progress of the step [3]. External rotation of the hip associated with increased internal rotation and hyperextension of the knee during the stance phase of walking [3] can result in abnormal forces acting on the bone-cement or cement-implant interfaces [13]. This can lead to recurrent subluxations generating shear stress which could lead to an early loosening of the prosthetic components. Once loosening has occurred, sustained stress forces can lead to fragmentation of the bone trabeculae, resulting in loss of bone volume and eventual migration of the prosthetic components.

In our case, a 72-year-old woman, presented with gradually worsening pain symptoms 10 years after TKA. Clinical and radiological evaluation confirmed massive aseptic loosening with migration of prosthetic components. The surgical strategy chosen was a one-stage revision and implantation of a rotating-hinge TKA (Nexgen RHK, Zimmer, Warsaw). Bone loss on the tibial side was compensated with a tantalum support. The revision to a constrained rotating-hinged TKA showed an excellent functional result three years following the surgery.

Given the rare nature of CMT disease and the lack of data in literature, recommendations for neurological disorders like myasthenia gravis or post-poliomyelitis patients can be taken into consideration [9]. Although CMT differs in pathophysiology from these neurological disorders, they share similar biomechanical consequences including lower limb muscle weakness, abnormal gait kinematics, and impaired joint stability. In such cases, given the multidirectional instability of these disorders, research primarily recommends the implantation of rotating-hinged TKA since standard condylar implants do not allow for proper stabilization of the joint, even when using constrained models [[14], [15], [16]]. While post-poliomyelitis sequelae are typically stable and result from prior flaccid paralysis, CMT is a progressive neuropathy characterized by distal symmetrical weakness and sensory loss. This distinction implies that CMT patients may experience ongoing deterioration in neuromuscular control and proprioception over time, further complicating long-term TKA outcomes and supporting the early use of more constrained implants, as standard condylar TKA designs are likely to fail within a short period under such biomechanical conditions [13,17]. In our case it is debatable whether the implantation of a semi-constrained TKA might have been more appropriate initially. In this context, “semi-constrained” TKA refers to implants that provide enhanced varus-valgus stability without a mechanical hinge, while “constrained rotating-hinged” TKA involves a linked mechanism designed to address severe instability in multiple planes. Additionally, it should be noted that CMT disease can show a very variable evolution with some patients being stable for an extended period of time and others showing rapid clinical deterioration with late onset. Therefore, it might be difficult to propose systematic treatment recommendations. However, as highlighted in a recent study based on the Australian registry, patients undergoing primary stemmed TKA have been shown to have lower rates of all-cause revision beyond 1.5 years (HR 0.84; 95 % CI 0.73 to 0.97; *P* = 0.01) and tibial component-only revision at all times (HR 0.47; 95 % CI 0.29 to 0.74; *P* = 0.001) [18], suggesting that the use of stemmed components may provide long-term benefits in carefully selected patients.

In conclusion, this case report highlights the importance for structured follow-up in patients suffering from CMT disease who have already undergone TKA in order to detect complications early. Based on the increased risk of early loosening, we recommend postoperative evaluations at 6 weeks, 3 months, 6 months, and 1 year, followed by annual clinical and radiographic follow-up thereafter, rather than limiting long-term surveillance to every 5 years. Loosening might occur at an early stage compared to the general population. It might be important to make neurologists and treating physicians aware of the potentially increased risk of loosening following total knee replacements in patients newly diagnosed with such a neurodegenerative condition, so that they can encourage them for an orthopaedic follow-up. In presence of major bone loss and instability, revision to a constrained rotating-hinged TKA might be an appropriate treatment.

## 4 Summary

Considering that Charcot-Marie-Tooth (CMT) disease can show a very variable evolution with patients being stable and others presenting worsening of muscle tone of the knee's dynamic stabilizing structures over time, no clear consensus regarding treatment recommendations of advanced knee osteoarthritis with this condition is available to our knowledge. Prosthetic components might be under increased strain in regard to the multiplanar instability and the altered gait biomechanics in this patients cohort. Regular follow-up of patients suffering from CMT disease and who underwent total knee arthroplasty (TKA) is important in order to detect complications early. The implantation of rotating-hinged TKA might be an adequate option as standard condylar implants do not allow for proper stabilization of the joint, even when using models with constraint. In our case, a 72-year-old woman, presented with gradually worsening pain symptoms 7 years after TKA. Clinical and radiological evaluation confirmed massive loosening with migration of prosthetic components. The surgical strategy chosen was a one-stage revision and implantation of a rotating-hinge TKA (Nexgen RHK, Zimmer, Warsaw). Bone loss on the tibial site was augmented using tantalum support. The revision to a rotating-hinged TKA showed an excellent functional result at three years after surgery.

## Author contribution

1) writing the paper.

2) writing review.

3) study concept and design.

4) data collection.

5) data collection.

6) study concept and design.

## Consent

Written informed consent was obtained from the patient for publication of this case report and any accompanying images. A copy of the written consent is available for review by the Editor-in-Chief of this journal on request.

## Ethical approval

Ethical approval was waived for this case report in accordance with the institutional policy for case reports involving fewer than five patients.

## Guarantor

Jaad Mahlouly.

## Research registration number

This case report does not involve a “First in Man” intervention and is therefore exempt from prospective registration in accordance with the Declaration of Helsinki 2013 and the editorial policies of IJS Case Reports.

## Funding

No funding was received for the preparation of this case report. The sponsors had no role in the collection, analysis, or interpretation of data; in the writing of the manuscript; or in the decision to submit the manuscript for publication.

## Conflict of interest statement

The authors declare that they have no known competing financial interests or personal relationships that could have appeared to influence the work reported in this paper.
